# The Ecology of Acidophilic Microorganisms in the Corroding Concrete Sewer Environment

**DOI:** 10.3389/fmicb.2017.00683

**Published:** 2017-04-20

**Authors:** Xuan Li, Ulrike Kappler, Guangming Jiang, Philip L. Bond

**Affiliations:** ^1^Advanced Water Management Centre, The University of Queensland, BrisbaneQLD, Australia; ^2^Centre for Metals in Biology, School of Chemistry and Molecular Biosciences, The University of Queensland, BrisbaneQLD, Australia

**Keywords:** sewer, concrete corrosion, acidophile, *Acidithiobacillus thiooxidans*, *Acidiphilium*

## Abstract

Concrete corrosion is one of the most significant problems affecting valuable sewer infrastructure on a global scale. This problem occurs in the aerobic zone of the sewer, where a layer of surface corrosion develops on the exposed concrete and the surface pH is typically lowered from around 11–10 (pristine concrete) to pH 2–4. Acidophilic microorganisms become established as biofilms within the concrete corrosion layer and enhance the loss of concrete mass. Until recently, the acidophilic community was considered to comprise relatively few species of microorganisms, however, the biodiversity of the corrosion community is now recognized as being extensive and varying from different sewer environmental conditions. The diversity of acidophiles in the corrosion communities includes chemolithoautotrophs, chemolithoheterotrophs, and chemoorganoheterotrophs. The activity of these microorganisms is strongly affected by H_2_S levels in the sewer gas phase, although CO_2_, organic matter, and iron in the corrosion layer influence this acidic ecosystem. This paper briefly presents the conditions within the sewer that lead to the development of concrete corrosion in that environment. The review focuses on the acidophilic microorganisms detected in sewer corrosion environments, and then summarizes their proposed functions and physiology, especially in relation to the corrosion process. To our knowledge, this is the first review of acidophilic corrosion microbial communities, in which, the ecology and the environmental conditions (when available) are considered. Ecological studies of sewer corrosion are limited, however, where possible, we summarize the important metabolic functions of the different acidophilic species detected in sewer concrete corrosion layers. It is evident that microbial functions in the acidic sewer corrosion environment can be linked to those occurring in the analogous acidic environments of acid mine drainage and bioleaching.

## Concrete Corrosion in the Sewer Environment

### Introduction

**FIGURE 1:**
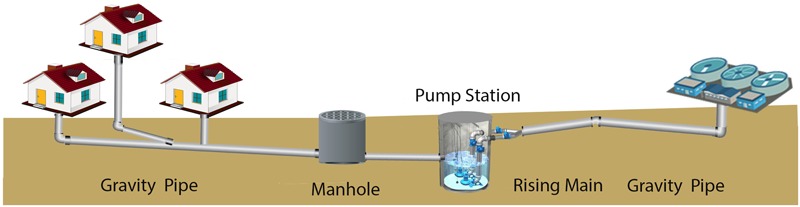
**Generalized overview diagram of sewer network infrastructure**.

**FIGURE 2:**
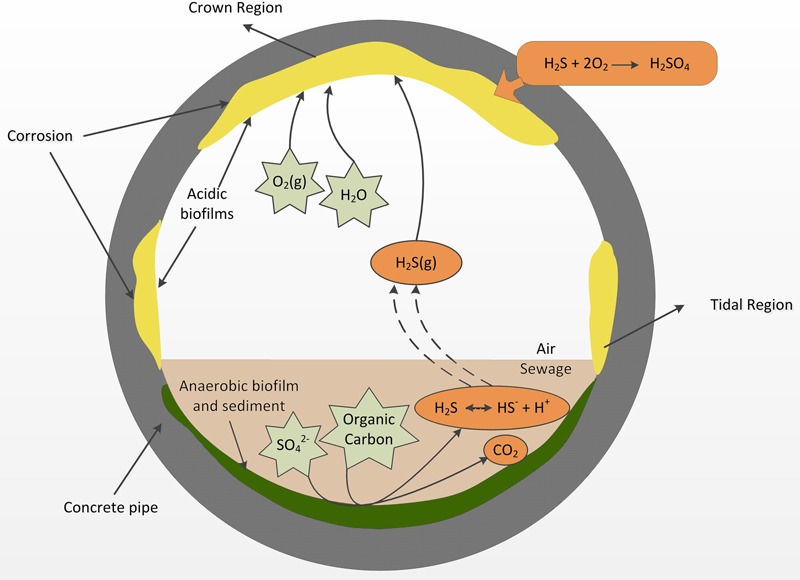
**A diagram of a cross section of a sewer gravity concrete pipe adapted from Hvitved-Jacobsen et al. (2013), summarizing the major processes that lead to acid formation in the aerobic biofilms and the onset of sewer corrosion**.

In developed countries sewer systems are implemented for the collection of wastewater and transportation of that to wastewater treatment facilities (**Figure 1**). These piping systems are extensive and are typically constructed of concrete. The total length of sewer in Australian is almost 117,000 km, and approximately 40% of this network is constructed from concrete. Through the piped connections from houses, drains, manholes, pump stations, and storm overflows, the collection system can be further divided into rising mains and gravity flow regions (**Figure 1**). The rising main sections are designed and operated to pump the sewage to a higher altitude and have no gas phase present within the pipes. In the gravity flow regions, the sewage flows due to gravity and these are always partially filled with sewage and thus have a gas phase (**Figure 2**). The broad range of pollutants and the high microbial presence within sewage is such that sewers function as ‘microbial reactors’ where substances are transformed and degraded based on their chemical and biological reactivity (Hvitved-Jacobsen et al., 2013). These microbial activities can lead to concrete corrosion, especially in gravity pipes. For many years the process of sewer concrete corrosion in the gravity sewer section was considered to be purely chemical (Olmstead and Hamlin, 1900) until it was revealed that the direct cause of sewer concrete corrosion, sulfuric acid, was generated biologically rather than chemically (Parker, 1945a,b, 1947).

Since those discoveries of Parker, the process has been termed microbially induced concrete corrosion (MICC) where microorganisms are involved in oxidation of hydrogen sulfide (H_2_S) that occurs in the headspace gas of the sewer (Parker, 1945a). Hydrogen sulfide production occurs in the sewer through the activity of sulfate reducing bacteria (SRB) mainly in the rising mains (Hvitved-Jacobsen et al., 2013) but also in the anaerobic sections of gravity pipes (**Figure 2**). After generation, the H_2_S is partitioned from the liquid into the gas phase in the gravity sewer pipes. The gaseous H_2_S can then partition into condensation layers on the gas-phase exposed walls of the concrete pipe (**Figure 2**). In those surface layers it is oxidized into sulfuric acid through the activity of aerobic sulfide-oxidizing microorganisms (Jiang et al., 2016). The sulfuric acid generated reacts with alkaline cement materials and produces gypsum and ettringite that have poor structural capacity and leads to weakened structure and eventual collapse of the concrete. Within the sewer, MICC often occurs in hot spots, which are located in regions just above the sewage flow level and at the ceiling of the headspace (Islander et al., 1991), respectively, referred to as the “tidal region” and the “crown region” (**Figure 2**). The tidal regions are frequently wetted by wastewater, while the crown region is exposed to the headspace sewer gas and only very occasionally (e.g., during flooding) contacts the wastewater (**Figure 2**).

Concrete corrosion is regarded as one of the most serious problems currently affecting the sewer infrastructure. Corrosion damage to sewers leads to premature replacement or rehabilitation which are estimated to cost several billion dollars every year globally (Pikaar et al., 2014; Jiang et al., 2015a). In addition, as hydrogen sulfide is toxic to humans and corrosive, the structural failure that emerges can cause damage to adjacent infrastructure, property and potentially to citizens (Jiang et al., 2015a).

When the MICC is well established, acidophilic sulfur-oxidizing microorganisms (ASOM) will be active in the extremely acidic conditions of the surface biofilms (around pH 2–4) (Joseph et al., 2012) and result in the rapid loss of concrete mass, which can be up to 10 mm year^-1^ (Zhang et al., 2008). Understanding details of the ecology of sulfide-oxidizing microorganisms, especially the ASOM associated with MICC is crucial for the control and mitigation of sewer corrosion. The molecular approaches to determine details of the microbiology have advanced greatly in the past decade and are providing revealing insight. This is especially with the application of high-throughput next generation sequencing (NGS) of small subunit rRNA gene amplicons (see further explanation in Acidophilic Microorganisms Detected in the Established Corrosion Layer) for community profiling and of whole community DNA for metagenomics (Cayford et al., 2012; Gomez-Alvarez et al., 2012). This, to the authors’ knowledge, is the first review to focus on acidophiles in the sewer corrosion process.

### Development of the Corrosion Layer

Concrete corrosion results from various abiotic and biotic processes, which are facilitated by the corrosive headspace gas present in sewers. During the initiation stage of concrete corrosion, abiotic processes such as carbonation (CO_2_) and H_2_S acidification reduce the surface pH of concrete from ∼13 to ∼9 (Joseph et al., 2012). After this initial abiotic acidification, biological processes, primarily sulfide oxidation, cause the direct formation of sulfuric acid which will further lower the concrete surface pH (Okabe et al., 2007).

#### Environmental Factors Involved in Sewer Corrosion

H_2_S is ubiquitous in sewers and it is believed at levels above 2 ppm it will cause sewer corrosion (O’Dea, 2007). However, H_2_S concentrations differ temporally and spatially (Jiang et al., 2014a; Wells and Melchers, 2015) with average concentrations seen to vary between a few ppm up to a few 100 ppm in most sewer systems, although in some extreme situations, concentrations as high as 550 ppm are detected (Wells and Melchers, 2015). Although there are volatile organic compound (VOC) emissions from the wastewater to the sewer gas, the concentrations are actually low at ppb to ppm levels or even less (Corsi et al., 1995; Quigley and Corsi, 1995; Chan and Hanaeus, 2006; Wu et al., 2006; Huang et al., 2012; Decottignies et al., 2013; Sivret et al., 2016). The smell of sewer gas is mainly caused by H_2_S (Jiang et al., 2015a; Sivret et al., 2016). This sewer gas phase H_2_S is a potential key energy source for chemolithotrophic sulfur oxidizing bacteria. Also, important for autotrophic microbial growth, elevated levels of CO_2_ (around 1.0%) have been detected in the headspace in various sewer field locations (Joseph et al., 2012).

Both abiotic and biotic processes heavily depend on the water content of the concrete, which is directly related to the relative humidity (RH) in sewer air. The sewer RH typically ranges between 60 and 100% (Ward et al., 2011; Wells et al., 2012). In addition, the tidal regions in sewer walls are frequently wetted by wastewater flooding, which will increase the water content of the concrete in those regions. At the high RH in the sewer pipes, moisture will condensate on the crown region, as the walls of sewer pipe usually have a lower temperature than the sewer atmosphere due to cooling from the surrounding soil. It is observed that high RH levels shorten the corrosion initiation and also facilitate the corrosion process (Jiang et al., 2014a, 2015b). Frequent wastewater flushing in the tidal regions, occasional flooding, high RH, and condensation water in the crown region facilitate the microbial colonization on the concrete surface as well as providing the essential medium for the chemical and biological sulfide oxidation reactions.

#### Conditions within the Corrosion Layer

**FIGURE 3:**
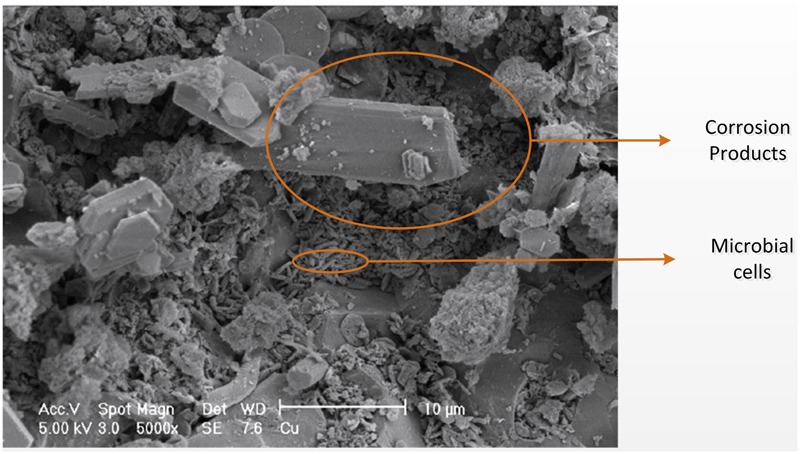
**Scanning electron microscopy image of the corroded concrete surface adapted from Cayford (2012).** Small elongated shapes are microbial cells, and angular crystal formations are sulfur-containing corrosion products (Cayford, 2012).

On the corroding concrete surface, a soft (cottage cheese like) moist layer forms comprising largely of crystalline gypsum and other sulfur species (discussed more below), within which microbial biofilms will exist (**Figure 3**). However, because of the minimal contact with wastewater and the paucity of nutrients innately present in concrete, the corrosion layer is likely very low in nutrients and organic carbon, especially on the concrete surface moving away from the tidal region. At the crown region, the main source of water is condensation, gas diffusion will provide volatile substrates and nutrients, and degrading microorganisms will also provide nutrient for growth. Although the corrosion is often reported to occur in the crown region, it is observed that more severe corrosion can occur in the tidal region where more frequent replenishment of nutrients, microbial inoculum, and moisture will occur in comparison to parts of the wall further away from that region (Jiang et al., 2014a).

**FIGURE 4:**
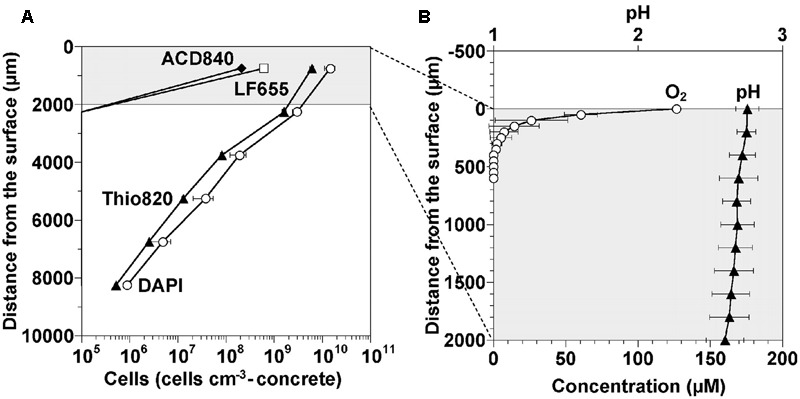
**Cell density, oxygen and pH profiles occurring in a corrosion layer developed on the surface of a cement coupon exposed to sewer conditions for 1 year (Okabe et al., 2007). (A)** Density profiles of cells detected in the gypsum layer. The non-specific DAPI stain and the specific probes Thio820, ACD840, and LF655 target *Acidithiobacillus* spp., *Acidiphilium* spp., and *Leptospirillum* spp., respectively. **(B)** Concentration profiles of dissolved oxygen and pH in the top 2,000 micrometers of the corrosion layer (Okabe et al., 2007) (reproduced with permission from American Society for Microbiology).

Oxygen diffusion into the corrosion layer is crucial for the activity of the aerobic microorganisms of MICC. Microsensors have been used to monitor the pH and oxygen changes that occur into the depth of a cement coupon corrosion layer (**Figure 4**) (Okabe et al., 2007). It was seen that the pH of the heavily corroded layer was around 2.6, down to a depth of 2 mm. Oxygen concentrations rapidly decreased with depth, accompanying a decrease of cell numbers (**Figure 4**), suggesting in this instance that because of the gas diffusion limitations (O_2_ and/or H_2_S), the microbes occurred mainly on the concrete surface where the biofilm was denser nearer the surface of the corrosion layer (Okabe et al., 2007).

**FIGURE 5:**
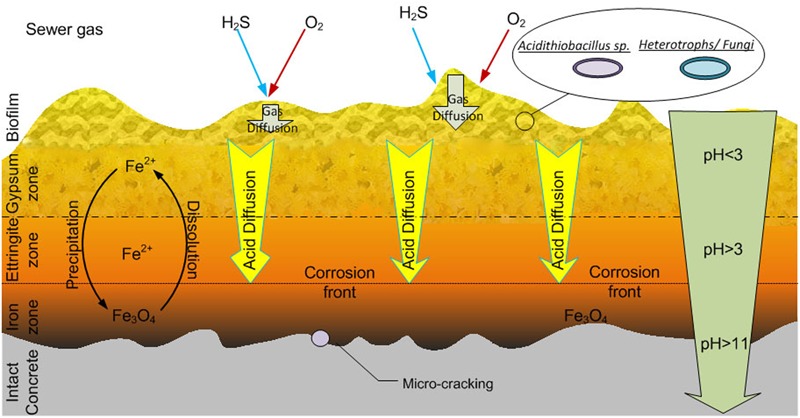
**A conceptual model for the sewer concrete corrosion layer as adapted from Jiang et al. (2014b).** See the text for description of the activities within the zones.

The activity of the biofilms in the top regions of the corrosion layer will produce acid (Okabe et al., 2007) which diffuses toward the intact concrete and reacts with the calcium silicates and calcium hydroxides of the concrete. Thus, the conditions within the corrosion layer vary with depth and are characterized by a large pH gradient (**Figure 5**). Gypsum (CaSO_4_) is formed preferentially in acidic conditions (at pH lower than 3), hence it is usually more prevalent nearer the surface of the corrosion layer (Mori et al., 1992). The gypsum within the corrosion layer reacts with calcium aluminate hydrate to form ettringite at higher pH, and thus the ettringite layer is beyond gypsum layer nearer the intact concrete. The depth of gypsum and ettringite layers depends on the sulfate concentration and pH. With increased acid production in the corrosion layer, ettringite may be converted to gypsum (Mori et al., 1992) and in some types of concrete, no ettringite is detected (Gutiérrez-Padilla et al., 2010). Unlike the original concrete matrix, gypsum and ettringite are non-structure-supporting materials and are both highly expansive (Monteny et al., 2000). The formation of gypsum and ettringite thus weakens the concrete structure and is believed to cause cracking of the concrete due to the expansive nature of the materials. Acid will diffuse into the corrosion layer to depths where oxygen diffusion is limited (**Figure 4**) and iron species, either from within the concrete or from iron rebar become dissolved and could transfer in the concrete pores and micro-cracks. Additional cracking of the concrete can also be caused by the rust precipitation in iron salt rich areas near the intact concrete (**Figure 5**) (Jiang et al., 2014b). Based on these observations, a conceptual model for the corrosion layer is proposed (**Figure 5**). The microbial biofilm is denser near the surface of the corrosion layer where the oxygen and H_2_S levels are highest (Okabe et al., 2007). Sulfuric acid is generated by ASOM in the biofilm and this diffuses through the corrosion layer toward the surface of the intact concrete and can penetrate this to a depth of at least 2 mm (Okabe et al., 2007). With the acid diffusion, the gypsum (pH < 3) and ettringite zones (pH > 3) are formed and as they are expansive, together with iron mineral precipitation, cause cracking of the intact concrete.

#### Temporal Succession of Microbes in the Corrosion Layer

Important microbial protagonists of the corrosion process are sulfur-oxidizing microorganisms as they accelerate the reduction of surface pH through the production of various sulfur compounds including sulfuric acid. As these processes lead to a gradual decrease of the concrete surface pH, microorganisms colonize the concrete and the composition of the microbial communities will change with time. Two types of microorganisms relevant for sewer corrosion are neutrophilic sulfur-oxidizing microorganisms (NSOM) and acidophilic sulfur-oxidizing microorganisms (ASOM) both of which use reduced forms of sulfur as energy sources, but respectively, have a preference for growth at a neutral or acidic pH (Islander et al., 1991; Roberts et al., 2002). After the pH of the sewer surface is abiotically reduced to 9, NSOM will colonize the surface and produce acid to eventually lower the pH to 5-3 (Roberts et al., 2002). The various dominant NSOM species detected include: *Thiothrix* spp., *Thiobacillus plumbophilus* (formally as *Sulfuriferula plumbophilus*) (Watanabe et al., 2015), *Thiomonas* sp., and *Halothiobacillus neapolitanus* (Okabe et al., 2007; Santo Domingo et al., 2011; Ling et al., 2015). *Thiothrix* spp. and *Thiomonas* spp. have the ability to grow chemoorganoheterotrophically or chemolithoautotrophically using either organic carbon or the inorganic sulfur species H_2_S, S_2_O_3_^2-^, and S^0^ as electron donors. In comparison, *Halothiobacillus* sp. are obligate chemolithoautotrophic sulfur-oxidizing bacteria. The initial activity of NSOM on the sewer surface provides suitable conditions for subsequent colonization by ASOM (Mild et al., 1983; Islander et al., 1991; Davis et al., 1998; Okabe et al., 2007; Jiang et al., 2015a).

## Acidophilic Microbial Communities in the Concrete Corrosion Layer

As mentioned above, there is a succession of microbial types involved in the concrete corrosion from the time of initial exposure, when the concrete surface is alkaline (pH 9–13), to when an established corroding concrete has developed and this is lowered to around pH 2–4. This review focuses on the acidophilic microorganisms of the established corrosion layer when the surface pH is acidic. Currently much importance is placed on the readily cultured bacterium *Acidithiobacillus thiooxidans* (formerly *Thiobacillus thiooxidans*) as a main biological component of sewer corrosion. Sulfur oxidation, as performed by these bacteria is undoubtedly a key metabolic activity, however, as revealed below, recent investigations of sewer corrosion ecology using culture independent detection techniques indicate that a diverse range of microorganisms is present. Recent studies performing microbial community profiling by NGS have advanced and are providing incredible new insight, even though molecular studies of the acidic concrete corrosion environment are still in their infancy.

### Acidophilic Microorganisms Detected in the Established Corrosion Layer

Conventionally, research of the acidophilic communities involved in MICC has been mainly based on culture-dependent methods, where microorganisms are grown from corrosion samples using various culture media (Davis et al., 1998; Keller and Zengler, 2004). Although these results provide insight into the cultivable microorganisms involved in MICC (Parker, 1945a,b; Harrison, 1984; Islander et al., 1991), they can be misleading due to the bias and limitations of the culture-based techniques. Only a small fraction (in the order of 1%) of bacteria present in the environment are readily obtained in pure culture in the laboratory (Staley and Konopka, 1985). The choice of growth substrates, supplements and conditions for cultivation heavily influences what will grow, and hence only a limited number of microorganisms are detected in individual culturing attempts.

The most typical genus of acidophilic microorganisms associated with biogenic acid production detected by culture dependent methods is *Acidithiobacillus* (Kelly and Wood, 2000), and the prominent species include *A. ferrooxidans, A. thiooxidans*, and *A. caldus* (Parker, 1947; Harrison, 1984; Islander et al., 1991; Cho and Mori, 1995; Davis et al., 1998; Nica et al., 2000; Hernandez et al., 2002; Yamanaka et al., 2002; Okabe et al., 2007). Acidithiobacilli are autotrophic sulfur-oxidizing bacteria (Nica et al., 2000) which is an essential advantage in an environment with low organic carbon availability and high levels of reduced sulfur compounds. *A. thiooxidans* is the most commonly cultured organism among *Acidithiobacillus* from sewer concrete corrosion (Islander et al., 1991).

Heterotrophic bacteria have been detected in sewer corroding concrete (Davis et al., 1998; Nica et al., 2000). In one study, these aerobic heterotrophs were detected to be in the range of 10^5^ cells/g of corrosion layer (Davis et al., 1998). Although the type of heterotrophs was not mentioned, this study reported significant quantities of heterotrophs in the sewer corrosion environment. That study also detected similar levels of NSOM but lower levels of ASOM in the corrosion layers. However, the possibility that these heterotrophs could be acidophiles was not examined as they were cultured on neutrophilic media (Davis et al., 1998). A subsequent study also isolated aerobic heterotrophs from corrosion layers (Nica et al., 2000). This includes isolates of the genera *Bacillus* and *Microbacterium* and of the species *Ochrobactrum anthropi*. These isolated heterotrophs were not shown to oxidize sulfur compounds during pure culture growth at or below pH 5 (Nica et al., 2000). Thus the nature of the growth of these heterotrophs in the acidic corrosion environment is not clear, although, it is suggested that these bacteria are scavenging organic compounds produced by growth of the NSOM and ASOM (Nica et al., 2000).

Heterotrophic fungi, have also been cultured from sewer corrosion samples (Cho and Mori, 1995; Nica et al., 2000), however, the fungi isolated in these studies were not formally identified. Fungi isolated by Cho and Mori (1995) were reported to grow at acidic conditions (pH 2.6), although neutral pH was the optimum for growth. Additionally, it is suggested that these fungi exist in a symbiotic relationship with ASOM by oxidizing H_2_S to thiosulfate and by utilizing organic compounds produced by the bacteria (Cho and Mori, 1995). Another study isolated a fungus from corroding concrete which was identified as a *Fusarium* sp. (Gu et al., 1998). However, *Fusarium* sp. are not reported to be acidophilic, although they are suggested to contribute to the corrosion process by production of organic acids (Gu et al., 1998).

The development of culture-independent methods has revolutionized studies of microbial ecology (Ward et al., 1990). These methods focus on analysis of DNA sequences of particular genes [amplified by the polymerase chain reaction (PCR)] and microbial community profiling is often performed by analysis of the small subunit rRNA genes. These are the 16S rRNA genes, for detection of bacteria and archaea, and the analogous 18S rRNA genes for detection of eukaryotes. NGS provides very efficient methodology to rapidly obtain thousands of gene reads that represent operational taxonomic units (OTUs) within the sample. Although, typically these are incomplete reads of a few hundred bases, so that the resolution for identification of the OTU is limited to the order, family, or genus level at best. More recently, whole or near complete genome sequences are obtained directly from various environmental samples through metagenomics (Hugenholtz and Tyson, 2008). This also provides basic community composition information, but more importantly individual genomic information is obtained from which functional capabilities of the microorganisms in the sample can be estimated. Presently there are few metagenomic studies of the sewer corrosion environment.

As mentioned, molecular based studies may utilize PCR amplification of genes from corrosion samples. However, it is reported that the success of the amplification can be variable, and this likely relates to the quality and quantity of DNA extracted from such samples (Cayford et al., 2012). The relatively low levels of microorganisms in the samples and the co-extraction of molecules inhibitory to the PCR amplification can limit these molecular based studies. Consequently, the non-cultivation techniques suffer from biases and the best studies will include multiple approaches, such as verifying sequencing results with FISH and other non-PCR based methods. The molecular based approach has been extremely enlightening and has led to significant new insights into the microbial communities involved in sewer concrete corrosion. Congruent with findings of culture dependent methods DNA sequencing directly from corrosion samples indicates that *Acidithiobacillus* spp. are often highly abundant in corrosion samples (Okabe et al., 2007; Satoh et al., 2009; Gomez-Alvarez et al., 2012; Ling et al., 2015; Jiang et al., 2016). According to culturing or by cloning and full 16S rRNA gene sequencing, these *Acidithiobacillus* spp. are likely *A. thiooxidans* (Parker, 1947; Harrison, 1984; Islander et al., 1991; Davis et al., 1998; Nica et al., 2000). However, there are instances where NGS analyses report low abundance or no detection of *A. thiooxidans* in sewer corrosion samples (Santo Domingo et al., 2011; Cayford et al., 2012).

High microbial diversity is not characteristic of acidic corrosion samples, as the communities can be dominated by few species (Pagaling et al., 2014). However, sequencing has revealed numerous different microbial types in these acidic samples. *Mycobacterium* spp. are often detected as abundant in acidophilic communities of sewer corrosion layers (Vincke et al., 2001; Okabe et al., 2007; Cayford et al., 2012; Pagaling et al., 2014). Cayford et al. (2012) investigated MICC communities in two sewers in Sydney and found *Acidiphilium* spp. and *Mycobacterium* spp. to be the most dominant acidophilic microbial groups across the 10 samples examined. In contrast, *Acidithiobacillus* spp. was prominent in only one sample and made up<3% of the total populations in all others. Previously, these acidophilic *Mycobacterium* were assumed to have a heterotrophic role in MICC, however, there are suggestions they may oxidize sulfur (Cayford et al., 2012), and recently an acidophilic *Mycobacterium* sp. has been observed to oxidize sulfur and produce acid (Kusumi et al., 2011). In Cayford’s study, wall and ceiling samples of the sewers were analyzed and in general higher microbial diversity was observed in the wall samples where significant proportions of *Xanthomonadales* spp., *Burkholderiales* spp., and *Sphingobacteriales* spp. were detected (Cayford et al., 2012). The *Xanthomonadales* are reported in other sewer corrosion environments (Satoh et al., 2009; Santo Domingo et al., 2011; Gomez-Alvarez et al., 2012), and are considered primarily as heterotrophs. Although the *Xanthomonadales* sequence from Cayford forms a phylogenetic cluster with no cultured representatives, the cluster affiliates with *Xanthomonadales* spp. which are shown to be capable of sulfur oxidation (Lee et al., 2007).

In a more recent study, crown samples were examined at six points located downstream from the outlet of a rising main (Pagaling et al., 2014). High acidity (averaging between pH 0.3–1.5) and low microbial diversity was reported, the lowest diversity coinciding with the more acidic samples. In five of the six crown samples *Mycobacterium* spp. dominated, being between 45 and 98% of the detected bacteria. *Acidithiobacillus* spp. were prominent in only two crown samples, and these being detected at between 28 and 36% of the total bacteria (Pagaling et al., 2014). *Methylacidiphilum* spp. were prominent in 2 of the crown samples. These are known acidophilic methanotrophs (Hou et al., 2008), and are likely utilizing methane that was detected in the headspace of that sewer.

On one occasion *Ferroplasma* spp. are reported in severely corroded concrete biofilms with an extremely low pH (Ling et al., 2015). *Ferroplasma* spp. are extreme acidophiles and versatile heterotrophs that can oxidize iron (Dopson et al., 2004), although they are not known for sulfur oxidation capabilities. Sequencing also detected an alga in an extremely acidic sewer corrosion layer (Pagaling et al., 2014). This was identified as *Cyanidium caldarium*, an organism detected in other acidic environments, and is thought to be growing heterotrophically in the sewer environment. Due to the depth of NGS and discovery by clone sequencing from sewer corrosion samples these molecular-based studies are detecting a wide range of microorganisms and many of those are considered to be primarily heterotrophic acidophiles and sometimes halotolerant (Okabe et al., 2007; Satoh et al., 2009; Santo Domingo et al., 2011). In addition to those mentioned above these proposed heterotrophs includes species of *Pseudoxanthomonas* (Satoh et al., 2009), *Ochrobactrum, Achromobacter, Azonexus, Acinetobacter*, and *Clostridium* (Okabe et al., 2007). In a study, sequencing a large number of cloned genes from crown and manhole samples, a wide range of taxa is detected (Santo Domingo et al., 2011). While considerable diversity can be detected in these studies of biofilms of sewer corrosion layers, often the matching reporting of environmental conditions is poor, even the pH may not be mentioned. These environmental factors vary considerably from site to site and are crucial for understanding the nature and extent of MICC and acidity that occurs. However, even from the imperfect data from these studies, a summary of core microorganisms important for acidic sewer concrete corrosion can be suggested (**Table 1**).

**Table 1:** Specific microorganisms detected in acidic concrete corrosion layers and their functions within sewers using culture independent methods.

Taxa	Frequency of detection^a^	Abundance in the microbial community	Environment	Potential functions
*Acidithiobacillus thiooxidans*	Nearly always	>95%	Manhole, H_2_S > 10 ppm and CO_2_ > 10,000 ppm, surface pH < 2 (Ling, 2013)	Sulfur-oxidation, Carbon-fixation, Acid production, EPS formation
		60%	Manhole, H_2_S 30 ± 20 ppm (Okabe et al., 2007)	
		N.r.^b^	Heavy corrosion sewer pipes (Vincke et al., 2001)	
		Up to 40%	Concrete crown downstream from a forced main with pH > 1 (Pagaling et al., 2014)	
		Around 80–95%	Corrosion chamber, 10 ppm H_2_S (Jiang et al., 2016)	
		Lower than 3%	Wall of sewer (Cayford et al., 2012)	
*Acidithiobacillus*	Often	1.5–9%	Corrosion chamber, 25 ppm H_2_S (Jiang et al., 2016)	Sulfur-oxidation, Carbon-fixation
*ferrooxidans*		N.r.	Corroded crown region of sewer (Hernandez et al., 2002)	Acid production, Iron-oxidation
		N.r.	Sewage treatment plant (Maeda et al., 1999)	EPS formation
*Acidithiobacillus caldus*	Often	35–50%	Corrosion chamber, 25 ppm H_2_S (Jiang et al., 2016)	Sulfur-oxidation, Carbon-fixation, Acid production, Iron-oxidation
*Acidiphilium* spp.	Often	Up to 70.2%	Ceiling of sewer (Cayford, 2012)	H_2_S-oxidation
		Up to 50%	Manhole of sewer system with severely corrosion (Ling et al., 2015)	Organic carbon utilization
*Mycobacterium* spp.	Often	17%	Corrosion chamber, 25 ppm H_2_S (Jiang et al., 2016)	Possible H_2_S-oxidation
		44%	Concrete crown downstream from a forced main with pH < 1 (Pagaling et al., 2014)	Organic carbon utilization
		43.3%	Ceiling of sewer in Melbourne (Cayford, 2012)	
*Ferroplasma* spp.	Rarely	N.r.	Severely corroded concrete in manhole (Ling et al., 2015)	Iron-oxidation, Organic carbon utilization
*Xanthomonadaceae*	Occasionally	over 5%	Manholes with greater than 10,000 ppm of CO_2_ and less than 10 ppm of H_2_S (Ling et al., 2015)	Organic carbon utilization
		18.8%	Ceiling of sewers in Gold Coast (Cayford, 2012)	Organic carbon utilization
Fungi	Occasionally	N.r.	Manhole (Santo Domingo et al., 2011)	H_2_S-oxidation, EPS formation

*^a^The frequency of occurrence of the taxa is determined as: *nearly always*, means the taxa are detected in nearly all the studies included in this table; *often*, means the taxa are present in more than 60% of studies in this table, *occasionally* their presence in less than 60% but more than 10% of publications. *Ferroplasma* spp. was only reported by Ling.*

*^b^N.r. indicates that the abundance in the microbial community was not reported.*

Cells have been detected directly in corrosion samples using probes targeting rRNA molecules by fluorescence in situ hybridisation (FISH). While this approach is likely to provide the most real quantitative estimations of cells in samples, unfortunately it is only very rarely applied to study concrete corrosion. FISH of concrete samples is conceivably a difficult procedure due to combination of relatively meager cell densities, variable cell activity, and interfering autofluorescence from mineral particles. Nonetheless, a successful FISH approach has been applied by performing the hybridisation in solution (rather than directly on samples dried onto microscope slides) and filtering portions of the solution onto black filters for microscopy (Hernandez et al., 2002). Total cells in corrosion samples of a sewer manhole and a pipe crown were determined to be 7.2 × 10^7^ cells/g of dry corrosion product and 4.5 × 10^8^ cells/g of dry corrosion product, respectively. *Acidithiobacillus* were detected at 9 and 13% of the total cells in the manhole and crown samples, respectively (Hernandez et al., 2002). In another study using FISH, corroded mortar samples were exposed in a sewer manhole for 1 year where a thick gypsum corrosion layer formed (10 mm) and pH in the range of 1.6–2.7 was detected (Okabe et al., 2007). Sequencing indicated *A. thiooxidans* was prominent, and FISH verified that *Acidithiobacillus* were 6.1 × 10^9^ cells/cm^3^ of corrosion layer, this being about 50% of the total cells. Both these studies verified *Acidithiobacillus* spp. as important acidophiles in MICC, however, use of a more extensive range of probes is required to obtain a more complete picture of community compositions. Both studies did use genus level probes for *Acidiphilium* and *Leptospirillum*, however, these were not detected, or detected at only small as amounts, in these corroded layers.

Another culture independent method for understanding microbial community composition and function is by metagenomic sequencing. This approach sequences the total genomic component and utilizes bioinformatics tools to directly access the genetic content of the entire community (Thomas et al., 2012). Compared with the 16S rRNA gene method, metagenomics not only reveals the taxonomic diversity of a community, but also allows the detection of functional genes and the compilation of complete and near complete genomes of the microorganisms within a sample. So far only one metagenomics study describing the composition of a concrete corrosion community in sewer crown has been performed (Gomez-Alvarez et al., 2012). The analysis used biofilm samples from the crown of a corroded concrete sewer pipe and showed that the dominant members were from a diverse range of aerobic and facultative anaerobic bacteria that included *Acidiphilium, Xanthomonas*, and even photosynthetic organisms of the Cyanobacteria, however, no hyper-acidophilic SOMs sequence was found (Gomez-Alvarez et al., 2012). The study highlights that the composition of species involved in concrete corrosion is very site specific.

### Microbial Activities and Interactions in the Corrosion Layer

In the sewer, H_2_S from the gas phase, will dissolve into the surface water of the corrosion layer to form bisulfide (HS^-^) and sulfide (S^2-^) ions. At pH 2–3 most will be in the form of H_2_S which will undergo chemical and biological oxidations, through a number of electron transfer steps to produce sulfuric acid, see below (Iron Oxidation). The oxidation of reduced sulfur compounds would be the major energy source in the oxidative corrosion layers of the dark sewer, an environment that is low in other inorganic or organic electron donating molecules. Reduced iron is another electron source that may be present mainly due to the reinforcing bar used in the concrete pipe. However, very little energy is gained from the oxidation of both reduced sulfur and reduced iron compounds and these organisms may utilize much energy for carbon fixation for growth (Johnson and Hallberg, 2008). Consequently, while the growth of these chemolithotrophs may be slow, their activity of substrate turnover (S and Fe) may be very high.

In relation to the limited types of electron donors, this would seemingly limit the range of metabolic guilds of microorganisms that proliferate in sewer corrosion layers. However, the diversity of microorganisms of corrosion communities can include chemolithoautotrophs, chemolithoheterotrophs, and chemoorganoheterotrophs (the later often referred to as heterotrophs). We have summarized the important metabolic functions of different acidophilic species found in the sewer corrosion layer of concrete (**Table 1**). Publications that focus on the detailed roles of acidophiles in the MICC layer are limited. Some functions can be inferred from the rare metagenomic studies of sewer corrosion. Often the microbial functions (presented in **Table 1**) are inferred from pure culture studies of related microorganisms obtained from other similar acidic environments, such as acid mine drainage and bioleaching (**Table 1**).

**FIGURE 6:**
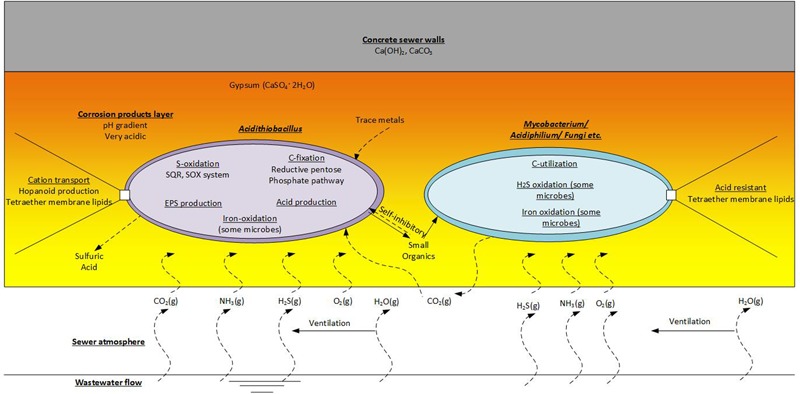
**Functions of the acidophilic microbes detected on the corrosion layer.** This is an imaginary microbial community that includes representatives of the chemolithoautrophic- *Acidithiobacillus* and the Chemoorganoheterotrophic- *Mycobacterium, Acidiphilium* and fungi etc.

Generally, *Acidithiobacillus* spp., as chemolithoautotrophs are a keystone group in the corrosion community. This group undertaking activities of sulfur-oxidation (and iron-oxidation for some species), carbon-fixation, production of extracellular polymeric substances (EPS) and the generation of sulfuric acid. The production of EPS would be an important activity that enables the biofilms in the corrosion layer to exist. As a major component of the biofilm matrix it would provide favorable habitat for the EPS producers and for other non-EPS producing microorganisms (Flemming and Wingender, 2010). However, there is diversity in microbial metabolisms in the corrosion layer as suggested by the diversity of microorganisms detected therein (Acidophilic Microorganisms Detected in the Established Corrosion Layer and **Table 1**). It is estimated that *Acidithiobacillus* spp. excrete as much as 20% of the CO_2_ they fix as small organic substances (Karavaiko and Pivovarova, 1973), and these excreted organic molecules can be inhibitory to their own growth (Okabe et al., 2007). Typically, the corrosion layer is expected to be low in organic carbon levels and the molecules excreted by *Acidithiobacillus* spp. would be important organic carbon source for energy and growth of the heterotrophic members of the microbial communities. Mutualistic relationships between *Acidithiobacillus* spp. and heterotrophs, that can degrade such inhibitory organic compounds, are proposed (Cho and Mori, 1995; Peccia et al., 2000). Therefore, simple roles for the proposed heterotrophs, such as *Mycobacterium* spp., *Xanthomonadales, Ferroplasma* spp., and fungi found in the sewer corrosion communities, would be the degradation of the secreted organic compounds and the production of CO_2_ (**Figure 6**). These activities would maintain favorable conditions for the growth of the chemolithoautotrophs, and enable their activities to continue and enhance the sewer corrosion. Some heterotrophs in this environment are also found to have the ability to oxidize H_2_S or iron. *Acidiphilium* spp. are sulfur oxidizing heterotrophs that are often detected in corrosion layer communities. Additionally, *Acidiphilium* are known to reduce ferric iron in anaerobic conditions (Hallberg and Johnson, 2001), although, that activity is likely negligible in the aerobic concrete corrosion layers. *Leptospirillum* spp. are also detected in corroding concrete. They are common in very acidic, iron-rich environments, and are obligate iron oxidisers (Hallberg and Johnson, 2001). Their presence in this environment may be related to the dissolution of iron bar used for sewer pipe reinforcement.

## Physiology of Acidophilic Microorganisms in Sewer Corrosion

The Baas-Becking hypothesis suggests ‘everything is everywhere, but the environment selects’ (De Wit and Bouvier, 2006). The typical low pH (<3) in advanced sewer concrete corrosion layer, accompanied with the limited availability of nutrients and organic carbon, pose a huge selective and evolutionary pressure on microorganisms (Sand and Bock, 1984; Islander et al., 1991; Okabe et al., 2007). To survive in such an environment, certain adaptations and physiological capabilities would be required.

### Adaptation to Low pH in the Concrete Corrosion Layer

Acidity is a main stress element in the concrete corrosion layer. Acidophilic microorganisms will try to maintain an intracellular pH that is near circumneutral, around pH 6.5–7.5. Many of the cells processes will be sensitive to pH, including those of DNA replication, protein synthesis and various enzyme activities (Baker-Austin and Dopson, 2007). Consequently, there is a large proton gradient across a thin cell envelope, that may be just 50–100 nm thick, and acidophilic microbes must have various adaptations to survive. The cell envelope in these organisms provides the primary and relatively impermeable barrier to protons (Baker-Austin and Dopson, 2007). While acidity is a stress, the large indigenous proton gradient would be utilized by acidophiles to generate ATP using membrane-bound ATPases (Johnson and Hallberg, 2008). However, this ATP production needs to be linked to proton efflux via electron transfer reactions (see Sulfur Oxidation) to avoid cellular acidification.

Modified membranes and membrane components are detected in these acidophiles. The archaea *Ferroplasma* have highly impermeable membranes composed of tetraether lipids (Batrakov et al., 2002). *A. ferrooxidans* is seen to reduce the pore size of an outer membrane porin in response increased acidity (Guiliani and Jerez, 2000), a mechanism also proposed to lower membrane permeability to protons. Another modification is to form an external capsule layer. The capsule layer is external to the membrane and consists of homogeneous polysaccharides and proteins (Schleifer and Kandler, 1972; Tuson et al., 2012). It is reported to help maintain cellular integrity and protect the organisms from various damaging external factors such as acidity and high concentrations of toxic heavy metals (Plante, 2000; Gao et al., 2014). *A. thiooxidans*, is reported to derive protection through the effect of a capsule layer. As the primary barrier to acid stress, this capsule layer is seen to thicken, in response to increasing acidity from pH 1.2–0.8 (Feng et al., 2015).

### Sulfur Oxidation

The oxidation of inorganic sulfur compounds (ISCs) would be the major energy conserving reaction in aerobic sewer corrosion environments. As mentioned above, this environment is comparatively low in other energy rich substrates. The oxidation pathways have been studied in only a few ASOM that are relevant to sewer concrete corrosion. Autotrophic acidophiles of *Acidithiobacillus, A. thiooxidans, A. caldus*, and *A. ferrooxidans* can oxidize a range of ISCs that includes H_2_S, elemental sulfur, tetrathionate (S_4_O_6_^2-^), trithionate (S_3_O_6_^2-^), and sulfite (SO_3_^2-^) (Johnson and Hallberg, 2008). The heterotrophic *Acidiphilium* spp. are also capable of sulfur oxidation in the presence of organic carbon (Hallberg et al., 2001). The current knowledge of ISCs oxidations by sewer acidophiles is drawn from pure culture studies, proven evidence of enzyme activities, and from speculative inferences through bioinformatic genome and metagenome analyses (Mangold et al., 2011; Cárdenas et al., 2016).

**FIGURE 7:**
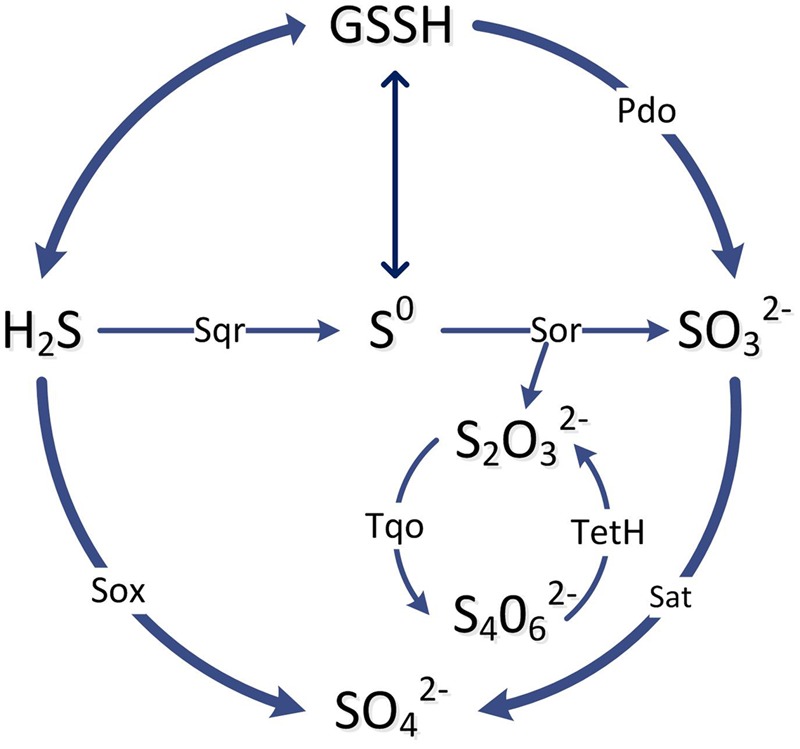
**A simplified scheme of the main pathways and enzymes involved in the oxidation of inorganic sulfur compounds by ASOM as explained in the text.** The enzymes in the pathway lines are: Sqr, sulfide: quinone oxidoreductase; Sor, sulfur oxygenase reductase; Pdo, persulfide dioxygenase; Sat, ATP sulfurylase; Sox, sulfur oxidation complex; Tqo, thiosulfate:quinol oxidoreductase; TetH, tetrathionate hydrolase.

Oxidation pathways of acidophiles in sulfur-rich environments have been reviewed recently (Dopson and Johnson, 2012). Various energy conservation pathways for the oxidation of ISCs to SO_4_^2-^ have been detected in sulfur oxidisers in general, and for ASOM, the pathways vary (Johnson and Hallberg, 2008). It is thought that the initial part of the scheme is similar for *A. ferrooxidans, A. thiooxidans*, and *Acidiphilium acidophilum* in that H_2_S and S^0^ enter the periplasm and are activated by membrane bound thiols to form persulfide groups (**Figure 7**), such as *S*-sulfanylglutathione (GSSH) (Rohwerder and Sand, 2003). The persulfide groups can release H_2_S which is oxidized to S^0^ and to sulfite by sulfide:quinone oxidoreductase (Sqr) and persulfide dioxygenase (Pdo), respectively. These oxidation steps pass electrons onto the cytoplasmic membrane quinone pool and then to various terminal oxidases to reduce oxygen and produce a membrane proton motive force (Quatrini et al., 2009). In *A. ferrooxidans* sulfite is then oxidized to sulfate for substrate level phosphorylation that is partly catalyzed by adenylyl-sulfate reductase (Sat) (**Figure 7**). In addition, an alternate tetrathionate intermediate (S_4_I) pathway occurs in *Acidithiobacillus* (Dam et al., 2007). As a central intermediate of H_2_S oxidation, thiosulfate is metabolized either through the sulfur oxidizing enzyme system (Sox) or the tetrathionate intermediate (S_4_I) pathway. In the S_4_I pathway thiosulfate is firstly oxidized into tetrathionate through thiosulfate:quinol oxidoreductase and then tetrathionate is hydrolysed by tetrathionate hydrolase to thiosulfate and other products (**Figure 7**) (Ghosh and Dam, 2009).

The genome sequence of *A. caldus* indicates it has some components of ISC metabolism similar to *A. ferrooxidans*, such as Sqr, truncated Sox, S_4_I and terminal oxidases (Wang et al., 2016). However, in contrast to *A. ferrooxidans*, it has genes coding the Sox enzyme complex to catalyze the oxidation of sulfide and thiosulfate to sulfate (**Figure 7**) (Mangold et al., 2011). Another difference in *A. caldus* is that S^0^ is oxidized to sulfite through the disproportionation reaction by sulfur oxygenase reductase (Sor) (**Figure 7**). The acidophiles, *A. thiooxidans* and *Acidiphilium* spp. may be similar to *A. caldus*, as genome analysis indicates they may also use the Sox pathway for oxidation of ISCs to sulfate (Rohwerder and Sand, 2007; Valdes et al., 2011). In addition, for the S_4_I pathway, a two-component system RsrS-RsrR has been reported to regulate the tetrathione intermediate pathway for thiosulfate oxidation in *A. caldus* (Wang et al., 2016).

*Mycobacterium* are primarily assumed a heterotrophic role in MICC. However, recently an acidophilic *Mycobacterium* sp. has been observed to oxidize sulfur and produce acid, although, there is no knowledge yet of the pathway involved (Kusumi et al., 2011). Other presumed heterotrophs may also partake in oxidation of ISCs. *Ferroplasma* spp. are not known for sulfur oxidizing activity (Tyson et al., 2004). However, they have recently been detected in acidic biofilms in sulfide-rich caves, and some genes for sulfur oxidation are found within *Ferroplasma* genomes such as sulfur oxygenase reductase (Sor) homologs (Jones et al., 2014). Additionally, as already mentioned (see Acidophilic Microorganisms Detected in the Established Corrosion Layer), *Xanthomonadales* spp., primarily considered as heterotrophs in the sewer corrosion environment, may also have ISC oxidation capabilities (Lee et al., 2007).

### Carbon Metabolism

In addition to the selection pressure presented by the acidic environment, the expected low level of nutrients in the corrosion layer is also likely to limit microbial growth and activity, and it could be expected that autotrophic microbes will be dominant.

Acting as a predominant producer in the acidic corrosion layer, *Acidithiobacillus* spp. fix inorganic carbon via the Calvin–Benson–Bassham (CBB) cycle (Calvin and Benson, 1948). Genes coding for enzymes of the CBB cycle are detected in *A. ferrooxidans, A. thiooxidans*, and *A. caldus* (Wood et al., 2004; Valdés et al., 2007). As well, activity of the key enzyme, ribulose bisphosphate carboxylase/oxygenase (RUBISCO), has been detected in these organisms (Shively et al., 1986; Esparza et al., 2010; You et al., 2011). In obligate autotrophs, RUBISCO may be packaged into organelles called carboxysomes, particularly during CO_2_-limiting conditions (Shively et al., 1998). Carboxysomes are considered to enhance CO_2_-fixation by increasing the concentration of HCO_3_^-^ in the organelle, and these have been detected in *Acidithiobacillus* spp. (Shively et al., 1998) and genes coding for carboxysomes are present in *A. ferrooxidans* (Esparza et al., 2010). For *A. ferrooxidans* there are three different gene clusters encoding RUBISCO in its genome. It is suggested that these are expressed and utilized in response to different environmental concentrations of CO_2_ (Yoshizawa et al., 2004). This may be important in sewer biofilms where microbial growth and diffusion restrictions within the corrosion layer could cause localized low levels of CO_2_ to occur.

In relation to carbon fixation, NAD(P)H, is an important cofactor in that process (Johnson and Hallberg, 2008). In the ASOM, the oxidation of ISCs provides electrons at a redox level high enough to drive NADPH formation directly. This means that the energy driven electron transfers (uphill) that are used by many autotrophs, are not required by various acidophilic sulfur oxidizing autotrophs (Johnson and Hallberg, 2008).

Other microorganisms in the corrosion layer, such as *Acidiphilium* spp., *Mycobacterium* spp. and fungi, are described as heterotrophic microorganisms that are scavenging organic compounds (Okabe et al., 2007). As mentioned, *Acidithiobacillus* spp. are known to produce organic exudates (Harrison, 1984) and these exudates, EPS and debris from dead cells will make up organic components of the biofilm matrix, which will enable growth and activity of the heterotrophic microorganisms therein. In turn the CO_2_ produced by the heterotrophs will be utilized by the chemolithoautotrophs (**Figure 6**). Additionally, it is well known that small organic molecules excreted by ASOM can be toxic and inhibit the growth and activity of those bacteria (Norris and Ingledew, 1992; Johnson, 1998). Thus the association of heterotrophic microorganisms is crucial for removal of these inhibitory organic compounds and enable the continued growth of the chemolithoautotrophs (Vincke et al., 2001). The importance of this in sewer corrosion is yet to be determined. There is also the potential that VOCs present in the sewer atmosphere could support the growth of heterotrophic microorganisms on corroding concrete. However, VOC are detected only at low concentrations in the sewer gas (Corsi et al., 1995; Quigley and Corsi, 1995; Chan and Hanaeus, 2006; Wu et al., 2006; Huang et al., 2012; Decottignies et al., 2013; Sivret et al., 2016).

### Iron Oxidation

Iron oxidation is considered less important with regard to concrete sewer corrosion as iron is generally in low concentrations in this environment. However, iron species, either in the cement or from the rebar imbedded to reinforce the concrete, become dissolved due to the diffusion of sulfuric acid and could transfer into the pores and micro-cracks of the concrete (Jiang et al., 2014b). Recently it is detected that concrete cracks can be caused by precipitation of iron oxides that occur ahead of the corrosion front. Thus, this activity can be an important part of the initial development of concrete corrosion (Jiang et al., 2014b).

For a few key reasons, microbial iron oxidation becomes more favorable at low pH and likely this occurs in the conditions of the corrosion layer. At low pH ferrous iron is highly soluble and the chemical oxidation of ferrous iron becomes very slow (Stumm and Morgan, 1970). Additionally, in the low pH high sulfate conditions, the redox potential of the Fe^2+^/Fe^3+^ couple is lowered and that of the oxygen/water couple is raised. Consequently, at pH 2 the net potential difference for the electron transfer from ferrous iron to oxygen is increased to 420 mV and the reaction is more thermodynamically favorable (Johnson et al., 2012).

Iron oxidizing microbes detected in sewer corrosion include *A. ferrooxidans* (Hernandez et al., 2002; Yamanaka et al., 2002; Okabe et al., 2007), *Ferroplasma* spp. (Ling et al., 2015) and *Leptospirillum* spp. (Okabe et al., 2007), the latter being an obligate iron oxidiser (Harrison, 1984). *A. ferrooxidans* is the most well studied of all acidophiles with regard to iron oxidation. Through oxidation of ferrous iron to ferric iron in *A. ferrooxidans* electrons are transferred to reduce oxygen and this utilizes protons within the cell. The membrane proton gradient that is generated is then utilized to transfer protons into the cytoplasm through the ATPase to synthesize ATP (Apel et al., 1980). The blue copper protein rusticyanin, located in the periplasm, is a key participant in the electron transfer process (Blake and Shute, 1994). Based on genetic and biochemical analysis, the model for the electron transfer indicates that, in the periplasm, electrons from ferrous iron oxidation first flow through a high-molecular-weight *c*-type cytochrome Cyc2 onto the protein rusticyanin. From rusticyanin, electrons then flow through the periplasmic dihemic *c-*type cytochrome Cyc1, and finally onto an *aa*3 type cytochrome oxidase on the inner membrane, passing electrons onto O_2_ which is reduced within the cell (Castelle et al., 2008). This is a well characterized respiratory system of bacterial acidophilic iron oxidation. However, it is seen that other acidophiles possess iron oxidizing mechanisms that are different to the *A. ferrooxidans* model.

In *Leptospirillum* spp. a periplasmic heme-containing protein cytochrome 579 is thought to be the key respiratory enzyme for iron oxidation (Ram et al., 2005; Blake and Griff, 2012). This was discovered in a groundbreaking study of an acid mine drainage biofilm using metagenomic and community proteomic analyses (Ram et al., 2005). It is proposed that cyt_579_ accepts electrons directly from ferrous iron, passing those onto other periplasmic cytochromes and then onto the inner membrane bound cbb3-type cytochrome oxidase for the intracellular reduction of oxygen (Ram et al., 2005). As for the *A. ferrooxidans* model, intracellular protons would be utilized in the process to maintain the membrane proton motive force. *Ferroplasma* spp. also use iron oxidation as an energy conservation process (Dopson and Lindström, 2004). Again, through metagenome analysis of acid mine drainage biofilm, a model is proposed for Fe(II) oxidation by *Ferroplasma* spp. (Allen et al., 2007). The model describes a blue copper sulfocyanin, a member of the protein family to which rusticyanin belongs, that transfers electrons to a cbb3-type terminal oxidase for the intracellular reduction of O_2_. At this stage details of other cytochromes or electron carriers involved in the respiration by *Ferroplasma* spp. are yet to be determined (Bonnefoy and Holmes, 2012).

In certain circumstances, it may be the case that acidophilic microorganisms in sewer corrosion are utilizing iron oxidation as the primary mechanism for energetic and growth requirements. *Leptospirillum* spp. have no other energy conserving capability, and *Ferroplasma* spp. is not known for sulfur oxidation (Tyson et al., 2004). Additionally, *A. ferrooxidans* may also perform iron oxidation. In this situation, electronegative potential is required to produce NAD(P)H, which are important reductants in various anabolic reactions of these organisms, including the role of carbon fixation in *Leptospirillum* spp. and *A. ferrooxidans*. Consequently, to produce these reducing equivalents, these iron oxidisers are forced to use energy and drive electrons in “uphill” reactions driven by the proton motive force (Bonnefoy and Holmes, 2012). In *A. ferrooxidans* this “uphill” electron transfer is seen to occur through the reverse functions of inner membrane enzymes, a *bc*_1_ complex and an NADH-Q oxidoreductase complex, in an ATP dependent reduction of NAD^+^ (Elbehti et al., 2000). Interestingly, the reduction of iron (III) is recently reported for some *Acidithiobacillus* spp. grown aerobically on elemental sulfur and for *Acidiphilium cryptum* grown micro-aerobically on glucose (Johnson et al., 2017). Although the reduction of iron (III) has not been reported in the oxic sewer corrosion layer, there is potential for this to occur in the iron transformations there.

### EPS Formation

In the sewer concrete corrosion layers, microbial cells are present in associations that are consistent of biofilm formations. However, to our knowledge, EPS is yet to be explicitly studied directly in these aerobic corrosion layers. There are numerous reports from the similar acid mine drainage environment, where the biofilm state is seen to enhance dissolution of the mineral ore (Sand et al., 2001). Similarly, it may be expected that EPS would provide important attributes for microbial activity and proliferation in the sewer corrosion environment. This could be important for initial attachment of cells from the sewage to the concrete. In the corrosion layer, the acidophiles will grow in favorable positions, i.e., access to substrates, oxygen and H_2_S and suitable pH (Okabe et al., 2007), with EPS being important for immobilizing cells. It may also provide other features typical of biofilms, such as stable conditions for moisture content and hold cells in close proximity for syntrophic interactions and DNA transfer (Gehrke et al., 2001).

There are few studies that characterize EPS from acidophiles that are known to inhabit sewer corrosion. For adhesion to pyrite and sulfur, *A. ferrooxidans* is seen to produce EPS that is hydrophobic and composed mostly of lipids and polysaccharides (Gehrke et al., 1998; Gehrke et al., 2001). Similarly, *A*. *thiooxidans* produces hydrophobic lipopolysaccharide EPS compounds that are important for its biofilm formation (González et al., 2012). *Acidithiobacillus* spp. are important components of sewer corrosion and likely their EPS production contributes to their proliferation in that environment. Genes and proteins for EPS production have been detected from *Leptospirillum* spp. in acid mine drainage biofilms (Ram et al., 2005). There it is suggested that certain members of the mixed culture biofilm are playing the important role of producing the binding matrix. It is seen that various acidophilic fungi including basidiomycetes, filamentous fungi, and yeasts will synthesize EPS (Mahapatra and Banerjee, 2013). Thus, fungi may also play a role for EPS production in the sewer corrosion environment.

## Summary and Future Directions

Sewer corrosion is universally accepted as a microbial corrosion process. The primary and direct cause is the biological sulfide oxidation by ASOM. While *Acidithiobacillus* spp. are often predominant in sewer concrete corrosion layers, recent studies using culture-independent methods identify diverse communities from these acidic biofilms that includes species such as *Acidiphilium* spp., *Mycobacterium* spp., *Xanthomonadales, Ferroplasma* spp. and fungi. In aerobic sewer biofilms, the ASOM play an important role in primary production through the oxidation of ISCs to conserve energy that is used for fixing carbon dioxide, for the synthesis of cell components and for production of extracellular materials (EPS). EPS would aid the attachment of the bacteria to the concrete surface and is the major component in microbial biofilms. Heterotrophic bacteria are an essential part of the sewer corrosion communities, as they degrade the extracellular organic molecules to support their growth, which also removes the presence of small organic molecules that may be toxic to ASOM.

Environmental factors such H_2_S, humidity, temperature, pH, and wastewater flooding play crucial roles on the corrosion process. It is essential that future molecular studies include more details of the environmental conditions that relate to corrosion. This is important so that valuable correlations can be made between the observed ecology and corrosion activity.

The corrosion process and core members of the microorganism community is summarized in this review. There is much interest to limit the activity of MICC to abate the destructive sewer corrosion process. Current control measures either try to lower the H_2_S concentration in the sewer and/or inhibit the activity of ASOM. To mitigate the generation and partition of H_2_S, liquid and gas-phase technologies using chemicals such as nitrates or iron salts are applied (Jiang et al., 2015a). Free nitrous acid (FNA), is an antimicrobial agent at ppm level, and is applied to limit the activity of SRB in the liquid phase of the sewer (Jiang et al., 2013) and has also been applied to directly inhibit the activity of ASOM in corrosion layers (Sun et al., 2015). Other technologies include the manufacture of corrosion-resistant concrete through the addition of admixtures, alternative binders or aggregates for new sewers. Additionally, existing sewer surfaces can be coated using epoxy, polymers, and various types of corrosion-resist materials (Bacelar-Nicolau and Johnson, 1999; Hewayde et al., 2007; Haile and Nakhla, 2008; De Muynck et al., 2009). However, it would be important to better understand how robust these applications are, such as the inhibitors and coatings. An improved understanding of how the microbial constituents of the sewer corrosion respond to these control measures is required. For example, this could determine the recovery potential or the survival and resistance of some species to the control measures.

## Author Contributions

The idea of this review paper was proposed by PB and GJ. The structure and whole paper was written by XL. PB made great contribution on the function of microorgansims. GJ helped with the process of concrete corrosion. UK helped with the details and language in this review paper.

## Conflict of Interest Statement

The authors declare that the research was conducted in the absence of any commercial or financial relationships that could be construed as a potential conflict of interest.
